# Evaluation of a Rapid Differentiation Test for *Mycobacterium Tuberculosis* from other Mycobacteria by Selective Inhibition with p-nitrobenzoic Acid using MGIT 960

**DOI:** 10.4103/0974-2727.72157

**Published:** 2010

**Authors:** Babita Sharma, Nita Pal, Bharti Malhotra, Leela Vyas

**Affiliations:** Department of Microbiology, SMS Medical College, Jaipur, India

**Keywords:** MGIT960, *mycobacterium tuberculosis*, non-tubercular mycobacterium (NTM), p-Nitro-benzoic acid

## Abstract

Tuberculosis is caused by *Mycobacterium tuberculosis* (M.tb) as well as Non-tubercular mycobacterium (NTM) with similar clinical presentation. Infections due to NTM are reported to have increased in the past few years. Growth of M.tb is inhibited by p-Nitrobenzoic acid (PNB), whereas, NTM are resistant. One hundred and nine isolates from various clinical samples were identified up to species level by their growth rate, pigmentation, and a battery of biochemical tests, including niacin accumulation, nitrate reduction, and heat-stable catalase (68°C) reactions. Para-nitrobenzoic acid (PNB) inhibition test was performed to differentiate between M.tb and NTM. PNB was added to the Lowenstein-Jensen (LJ) medium and BACTEC™ MIGIT (Mycobacteria Growth Indicator Tube)960 medium to a final concentration of 500 μg/ml. All the M.tb isolates, including *Mycobacterium tuberculosis* H37Rv (standard strain), were inhibited by PNB on both LJ and MGIT 960. Of the NTM isolates, all were resistant to PNB on MGIT 960 and on LJ PNB, except one isolate of *Mycobacterium marinum* that was resistant to MGIT 960 PNB, but was susceptible to LJ PNB. The reporting time for M.tb ranged from 4–11 days (median 5.9 days) by MGIT 960 and for NTM it was 2–10 days with an average of 4.5 days. This study was carried out to establish the accuracy and efficiency of MGIT 960 PNB and to differentiate between M.tb and NTM.

## INTRODUCTION

Of all infectious diseases tuberculosis is the leading killer and is endemic in India. Coinfection with Human immunodeficiency virus (HIV) increases the morbidity due to the disease. Tuberculosis in humans is primarily caused by *Mycobacterium tuberculosis* (M.tb), however, infections due to Non-tubercular mycobacterium (NTM) are reported to have increased in the past few years.[1,2] In immunocompetent hosts NTM primarily occurs as a respiratory pathogen, especially in individuals with chronic obstructive pulmonary disease and may cause pulmonary fibrosis or cavitary lung disease,[3] while in immunocompromised hosts it produces systemic bacterial infection.[4,5] Patients with M.tb infection are easily and successfully treated with primary antituberculosis drugs, while the therapy of the disease caused by NTM is often long, strenuous, and not always successful.[6] Thus, it is important to differentiate infections due to M.tb and NTM at the early stage of the disease.

Differentiation of M.tb from NTM can be done by colony morphology, smear microscopy, biochemical reactions or molecular methods, which are time-consuming and cumbersome. To overcome this, various selective inhibition tests have been described, which differentiate M.tb from NTM. Agents such as Hydroxylamine hydrochloride (HA), 8-azaguanine,[7] sodium salicylate, p-nitrobenzoic acid,[8] p-nitro-α acetylamino β hydroxypropiophenone (NAP),[9,10] nitroxoline,[11] propylene glycol,[12] and so on, selectively inhibit the growth of M.tb. Out of these methods many of them are technically demanding and time- consuming, while others are difficult to interpret and few require specialized reagents that are difficult to procure.

For rapid differentiation of mycobacterium various automated and semi-automated methods are available, which use liquid media and one of the selective inhibiting agents. These methods are BACTEC 460 TB system (Becton Dickinson Microbiology Systems, Sparks, Md.), MB/BacT system (Organon-Teknika, Durham, N.C.), ESP culture system II (AccuMed International, Westlake, Ohio), BacT/ALERT 3D system (Bio-Merieux, Durham, N.C.), and MGIT 960 (Becton Dickinson Microbiology Systems, Sparks, Md.). The BACTEC 460 TB system differentiates M.tb from NTM by selective inhibition, using p- nitro-α acetylamino β hydroxypropiophenone (NAP test), within four to six days. Many molecular methods like multiplex PCRs, DNA probes, and/or DNA sequencing have made the characterization of NTM species less ambiguous, more precise, rapid, cost-effective, and can be used directly on clinical samples. Recently, excretory proteins such as MPB64 and MPT63 have shown potential for differentiating MTB and NTM with high accuracy.

The MGIT 960 system is a fully automated continuous monitoring system, designed for the rapid detection of mycobacterium in all types of clinical samples, except blood and urine. The system includes a liquid culture medium, growth supplement, and an antibiotic mixture. The working principle of the MGIT system is based on oxygen quenching fluorescent technology to detect the amount of oxygen consumption induced by the growing microorganisms. Para nitrobenzoic acid (PNB) is a selective inhibitor of M.tb. It is readily available and various reports have been published in literature about its importance in differentiating M.tb and NTM. It is a simple inhibitory test, well adapted for MGIT 960, and provides highly accurate and rapid results by employing many mycobacterial species.

The objective of the present study was to differentiate the mycobacterium species isolated from different clinical samples of human patients suspected of having tuberculosis. These isolated species were characterized as M.tb and NTM by the MGIT 960 PNB test, LJ PNB test, and the conventional biochemical tests. This study was carried out to establish the accuracy and efficiency of MGIT 960 PNB, to differentiate M.tb and NTM.

## MATERIALS AND METHODS

The study was conducted on 109 isolates obtained from various clinical samples. After isolation in MGIT 960 tubes the isolates were sub-cultured on LJ media for further identification. The isolates were inoculated into the LJ medium and MGIT 960 medium containing PNB. The mycobacterial isolates were identified up to species level by their growth rate, pigmentation, and battery of biochemical tests including niacin accumulation, nitrate reduction, and heat-stable catalase (68°C) reactions.[13,14]

Standard strains included in the study were *Mycobacterium tuberculosis* H37Rv, *Mycobacterium avium* (JALMA), and *Mycobacterium intracellulare* (JALMA).

### Para nitrobenzoic acid test

Media: PNB was incorporated into the LJ medium and into the MGIT 960 medium at a final concentration of 500 μg/ml. This concentration of PNB was selected on the basis of previous reports.[15,16]

Inoculum preparation: The inoculum was prepared from the positive MGIT culture tube within one to five days after it flagged positive. An undiluted inoculum was used from day one and two MGIT positive culture tubes, while from the day three to five tubes, the suspension was diluted 1:5 with sterile saline. This was considered as the work suspension.

Inoculation: For each isolate two MGIT tubes were used, one containing plain media and the other containing 500 μg/ml of PNB. These tubes were inoculated with 500 μl of 1:5 dilution of work suspension. The same isolate was inoculated with 5 μl of work suspension on plain LJ media and LJPNB media.

Incubation: The MGIT 960 tubes were kept in a two-tube AST set carrier with the growth control tube in the left position and the drug containing tube in the right. The AST set carrier was loaded into the MGIT 960 instrument after scanning its barcode, which assigned its station. The LJ media were incubated at 37°C and were read daily for five days and then every week for four weeks.

Interpretation: The MGIT 960 instrument electronically monitors the culture tubes every hour and detects algorithms based on the assessments of changes associated with microbial growth. The instrument then automatically interprets these results and reports as susceptible or resistant in 4 to 21 days. When the growth index of the tube containing PNB is more than 100 it is reported as resistant, that is, NTM, and when less than 100 as sensitive, that is, M.tb. In the case of *Mycobacterium cheloni* and *Mycobacterium fortuitum* the system removed the tubes as they had given invalid results, because they are rapid growers and the protocol in the instrument is set for four days. From these tubes, smears are prepared, to determine the presence of acid fast bacilli by using the ZN stain and they are also sub-cultured on BHI agar to rule out contamination. These isolates are confirmed by biochemical reactions.

On LJ media the isolates were considered to be resistant, while LJ media containing PNB presented growth patterns similar to those in the control media.

## RESULTS

The results of the PNB growth inhibition tests in the LJ and MGIT 960 systems, using mycobacterial isolates, are presented in Table 1. All the mycobacterial isolates, including standard strain *Mycobacterium tuberculosis* H37Rv, were inhibited by PNB on both LJ and MGIT 960. Of the NTM isolates, all were resistant to PNB on MGIT 960 and on LJ PNB, except for one isolate of *Mycobacterium marinum*, which was resistant in MGIT 960 PNB, but susceptible in LJ PNB.

**Table 1:** Evaluation of PNB inhibition test on LJ medium and the MGIT 960 system

S. Species No.	No. of Strains/isolates	Niacin		Nitrate		Catalase		MGITPNB		LJPNB	
		P	N	P	N	P	N	S	R	S	R
M. tuberculosis H_37_Rv	1	1	0	1	0	0	1	1	0	1	0
M.avium (JALMA)	1	0	1	0	1	1	0	0	1	0	1
M. intracellulare (JALMA)	1	0	1	0	1	1	0	0	1	0	1
M.tuberculosis	78	78	0	78	0	0	78	78	0	78	0
M. scrofulaceum	1	0	1	0	1	1	0	0	1	0	1
M. xenopi	5	0	5	0	5	5	0	0	5	0	5
M. fortuitum	8	0	8	8	0	8	0	0	8	0	8
M. chelonae	6	0	6	0	6	6	0	0	6	0	6
M. simiae	2	2	0	0	2	2	0	0	2	0	2
M. kansasii	2	0	2	2	0	2	0	0	2	0	2
M.gordonae	3	0	3	0	3	3	0	0	3	0	3
M. marinum	4	0	4	0	4	0	4	0	4	1	3

P - positive, N - negative; S - Sensitive, R - resistant

The reporting time for M.tb ranged from 4–11 days (median 5.9 days) by MGIT 960, and 2–10 days by NTM, with an average of 4.5 days.

## DISCUSSION

Tuberculosis is caused by M.tb as well as NTM; although clinically they produce a very similar disease. It is important to identify mycobacterium up to the species level, as it provides a great deal of useful information on epidemiology and facilitates the successful treatment of patients.

Presumptive differentiation between M.tb and NTM can be made by growth rate, colony morphology, and pigmentation on LJ media, and by microscopic observation of cord formation on Zeihl Neelson staining[17,18] of a positive culture. This is not a conclusive method, as some M.tb do produce pigmentation and some NTM also show cord formation. Definitive identification can be made by using conventional biochemical tests, such as, niacin accumulation, nitrate reduction, heat-stable catalase (68°C) and other tests that are time consuming and cumbersome. In MGIT 960 the colony morphology cannot be visualized, so presumptive identification requires subculturing on solid media, which takes another three to four weeks. It improves the isolation, but delays the diagnosis. Therefore, there is a need to establish a rapid, reliable, and accurate method to differentiate M.tb and NTM by MGIT 960.

In the present study we processed 112 isolates of mycobacterium including three standard strains. All the 78 clinical isolates of M.tb were susceptible on MGIT 960 and LJ media containing PNB. All the 31 clinical isolates represented by eight NTM species were found to be resistant to PNB in the MGIT 960 medium. Out of these 31 isolates of NTM, all except one isolate of *Mycobacterium marinum*, were resistant to LJ PNB.

In our study there was 99.05% accuracy between MGIT 960 PNB and LJ PNB in differentiating M.tb and NTM. Giampaglia *et al*.[16,19] have reported 99.4% accuracy between MGIT PNB and LJ PNB when using manual MGIT and 98.7% when using MGIT 960 PNB and LJ PNB. The results are also in agreement with the results of conventional tests.[8,15,20]

The mean reporting time was 5.9 days and 4.5 days for M.tb and NTM, respectively, in the present study, while Giampaglia *et al*.[19] reported 6.3 days for M.tb and 2.5 days for NTM by MGIT 960.

Our figures indicated that the MGIT 960 PNB inhibition test was a rapid and specific method for the primary differentiation of M.tb from NTM. By this method the presumptive identification of M.tb and NTM could be done in 2–11 days after primary growth detection. In contrast the most widely used niacin production test requires a culture more than three weeks old and heavy mycobacterium growth to produce a detectable amount of niacin.

Our study demonstrates that the MGIT 960 PNB test for selective inhibition of M.tb is rapid and highly accurate. The average reporting time for M.tb was 4–11 days as compared to 28 days for the LJ PNB test and about one to two months when using the conventional biochemical test. As this is a growth inhibition test the growth in the MGIT 960 PNB vials should always be compared with those in the control vial without PNB.

Differentiation of M.tb from NTM can also be done by various molecular methods. The limitation of our study was that these facilities were not available at our laboratory during the course of study.

Thus, we conclude that in settings where molecular diagnostic facilities are not available the presumptive identification of M.tb can be performed for each isolate by using the PNB inhibition test as soon as it is recovered from the MGIT 960. If the isolate is MGIT PNB, sensitive drug sensitivity should be performed.
